# Comprehensive Analysis for GRF Transcription Factors in Sacred Lotus (*Nelumbo nucifera*)

**DOI:** 10.3390/ijms23126673

**Published:** 2022-06-15

**Authors:** Gui-Zhen Chen, Jie Huang, Xiao-Qin Zhou, Yang Hao, Jin-Liao Chen, Yu-Zhen Zhou, Sagheer Ahmad, Siren Lan, Zhong-Jian Liu, Dong-Hui Peng

**Affiliations:** Key Laboratory of National Forestry and Grassland Administration for Orchid Conservation and Utilization at College of Landscape Architecture, Fujian Agriculture and Forestry University, Fuzhou 350002, China; cgz1020@126.com (G.-Z.C.); 13530401396@163.com (J.H.); sczhouxiaoqin@163.com (X.-Q.Z.); hobart2016hy@outlook.com (Y.H.); fjchenjl@126.com (J.-L.C.); zhouyuzhencn@163.com (Y.-Z.Z.); sagheerhortii@gmail.com (S.A.); lkzx@fafu.edu.cn (S.L.)

**Keywords:** sacred lotus, GRF family, flower development, subcellular localization, transcription factor

## Abstract

Sacred lotus (*Nelumbo nucifera*) is an aquatic perennial plant with essential food, ornamental, and pharmacological value. Growth-regulating factor (GRF) is a transcription factor (TF) family that plays an important role in regulating the growth and development of plants. In this study, a comprehensive analysis of the GRF family in *N. nucifera* was performed, and its role in *N. nucifera* development was studied. A total of eight GRF genes were identified in the *N. nucifera* genome. Phylogenetic analysis divided the 38 GRF genes into six clades, while the NuGRFs only contained five clades. The analyses of gene structures, motifs, and cis-acting regulatory elements of the GRF gene family were performed. In addition, the chromosome location and collinearity were analyzed. The expression pattern based on transcriptomic data and real-time reverse transcription-quantitative PCR (qRT-PCR) revealed that the GRF genes were expressed in multiple organs and were abundant in actively growing tissues, and the expression levels decreased as the age of *N. nucifera* increased. Then, 3D structures of the NuGRF proteins were predicted by homology modeling. Finally, the subcellular localization of GRF1 was ascertained in the tobacco leaf through a vector. Therefore, this study provides a comprehensive overview of the GRF TF family in *N. nucifera*.

## 1. Introduction

Transcription factors (TFs) are DNA-binding proteins that can interact specifically with cis-acting elements in the promoter regions of eukaryotic genes and are major regulators of gene expression [1,2,3]. They play an important role in plant growth and development, fruit ripening, senescence, and response and adaptation to various stresses and defense responses (ref). Growth-regulating factor (GRF) is a plant-specific TF that plays a vital role in regulating plant growth and development [1]. There are two characteristic conserved domains, QLQ and WRC, in the N-terminal region of the protein-coding sequence [4]. The QLQ domain can interact with GRF interacting factors (GIF), and the obtained compounds can be used as transcriptional coactivators [5]. The WRC domain contains a nuclear localization signal (NLS) that can target the transcription factor to the nucleus and a zinc finger structure that binds to DNA [2]. In addition, some GRF transcription factors may also contain non-conservative domains, such as TQL, FFD, and GGPL, in the C-terminal [6]. Since the discovery of the first GRF in rice [7], a large number of GRF TFs have been isolated and identified [7,8,9,10,11,12,13], e.g., the model plant *Arabidopsis thaliana* has 9 GRFs [3], the tomato has 13 [14], *Brachypodium distachyon* has 10 [4], rice has 11 [9], and tobacco has 25 [15].

The GRF gene family plays a crucial role in the formation of tissues and organs during various plant biological processes, especially during early plant growth. These include leaf development [16], root growth [2], stem elongation [7], flower organ maturation [17], seed formation [18], hormone signaling [19], etc. The GRF family is strongly expressed in root tips, flower buds, and young leaves. The overexpression of *BnGRF2a* of *Brassica napus* can increase the seed oil yield by 50%, increase leaf extension by 20%, and increase photosynthetic efficiency by 40% [20]. *AtGRF1*, *AtGRF2*, and *AtGRF3* of *A. thaliana* are involved in leaf growth and development, and overexpression of *AtGRF1* and *AtGRF2* cause leaf and cotyledon enlargement and delayed flowering in *A. thaliana.* Overexpressed or disrupted *AtGRF5* affects the size of *A. thaliana* leaves, and compared with other members of the *Arabidopsis* GRF gene family, *AtGRF5* plays a more important role in the growth and development processes [16]. *AtGRF8* is involved in floral organ development in *A. thaliana* [16]. *AtGRF9* negatively regulates leaf growth by inhibiting cell proliferation in the leaf primordia [21]. In addition, the over-expression of the *BnGRF2a* gene in *A. thaliana* promotes gynoecium elongation, resulting in a low self-crossing rate close to that of sterility [20]. Moreover, the GRF gene family also regulates the development of cotyledons, flowers, and fruits in tomatoes [14].

*Nelumbo* belongs to Nelumbonaceae, which only comprises two species: *N. nucifera* Gaertn. and *N. lutea* Willd. [22]. *N. nucifera* is mainly distributed in China, India, Japan, Australia, and Iran [23]. *N. nucifera* is domesticated as a food and ornamental crop for its seeds, rhizomes, and flowers [24]. Since the genomes of *N. nucifera* were resolved [25,26], a large number of TFs have been isolated and identified, such as MADS-box [26], WRKY [27], R2R3-MYB [28], GRAS [29], etc. However, the GRF gene family has not been identified in *Nelumbo*. In this study, the GRF gene family of *N. nucifera* was identified on the basis of its complete genome sequence. A comparative analysis and an assessment of the phylogenetic relationship between *N. nucifera*, *A. thaliana*, *Nymphaea colorata*, and *Oryza sativa* were performed. Moreover, the genomic distribution, gene structure, and conserved motifs were identified. Then, the expression pattern, homology modeling, and subcellular localization of the GRF gene family in *N. nucifera* were further analyzed. The genome-wide identification and expression analysis of the GRF gene family will provide a basis for further research on the GRF gene family’s functions in the growth and development of *N. nucifera*.

## 2. Results

### 2.1. Identification of GRF Genes

To identify the GRF genes in *N. nucifera*, *A. thaliana* and *O. sativa* were used as query sequences, and CD-search and SMART were used to predict whether the candidate genes contained typical QLQ and WRC domains. Eight non-redundant protein sequences containing typical QLQ and WRC domains were obtained and renamed *NuGRF1~NuGRF8*. The number of GRF genes was less than that of most plants, such as *A. thaliana* (9), *O. sativa* (12), and *Nymphaea colorata* (9). The number of amino acids ranged from 349 to 604 aa, with the smallest one belonging to *NuGRF6* and the largest belonging to *NuGRF1* (Table 1). The isoelectric point (PI) of the GRF genes was 6.36–8.95; the molecular weight was between 535 and 65,565 KD; and the instability index was 55.27–65.39. The aliphatic index of the GRF genes ranged from 47.28 to 65.71. The results of subcellular localization prediction showed that eight NuGRFs were located in the nucleus.

### 2.2. Phylogenetic Analysis

**Figure 1:**
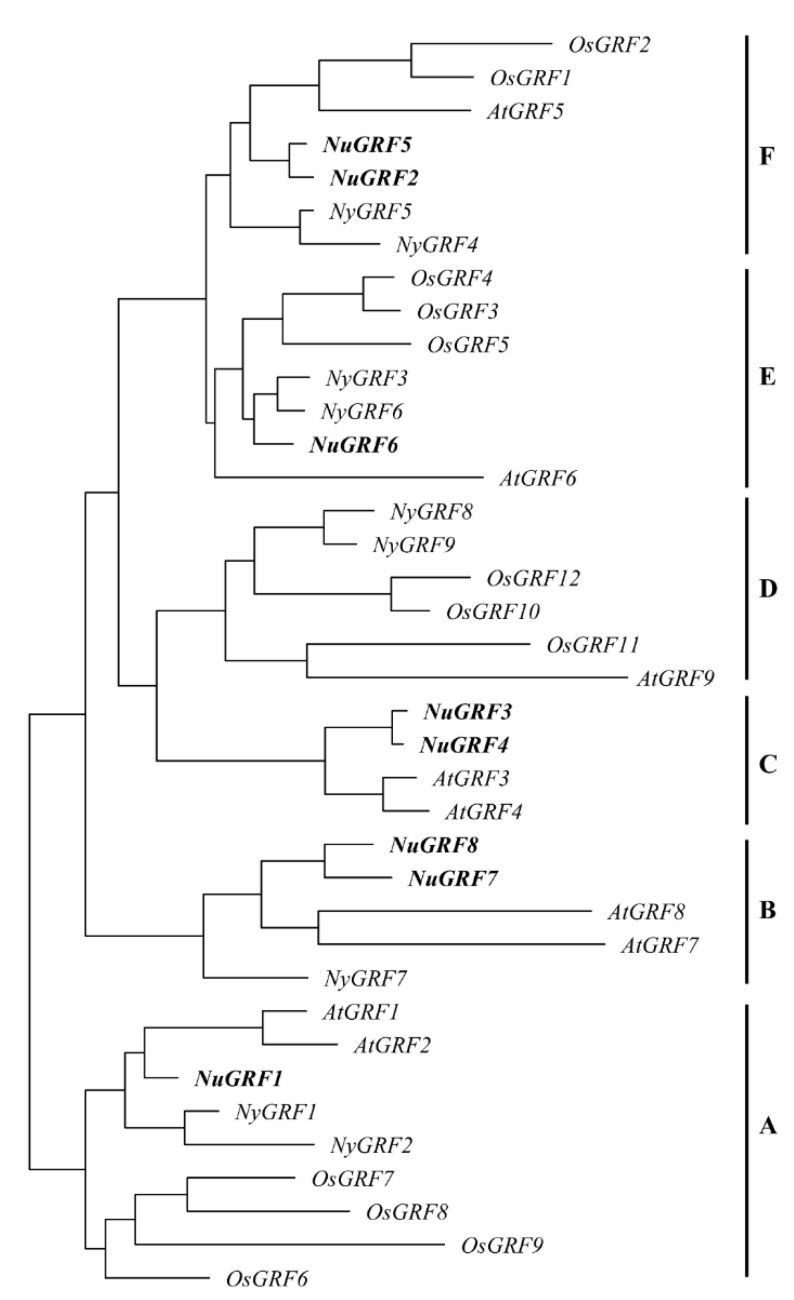
Phylogenomic tree of GRF genes. The genes beginning with “Nu” represent the genes of *N. nucifera*, “Os” represents the genes of rice, “Ny” represents the genes of *Ny. colorata*, and “At” represents the genes of *A. thaliana.* “A” represents Clade A, “B” represents Clade B, “C” represents Clade C, “D” represents Clade D, “E” represents Clade E, “F” represents Clade F.

To better verify the evolutionary relationships of GRF genes, a phylogenetic tree was constructed by comparing the identified *A. thaliana*, *O. sativa*, and *Ny. colorata* protein sequences. The result showed that the 38 GRFs were divided into six major clades, named as clades A~F (Figure 1). Clade A comprised two AtGRFs, two NyGRFs, one NuGRF, and four OsGRFs. It could be divided into two subclades, with the four OsGRFs clustered together. Clade B had only eudicots and the Nymphaeaceae species, containing one NyGRF, two AtGRFs, and two NuGRFs. Clade C had only eudicot species, containing two NuGRFs and two AtGRFs. Clade D had no NuGRFs, and it contained two NyGRFs, three OsGRFs, and one AtGRF. Clade E was composed of one AtGRF, one NuGRF, two NyGRFs, and three OsGRFs. Clade F comprised one AtGRF, two NuGRFs, two NyGRFs, and two OsGRFs. Our results showed that the NuGRFs were more closely related to *A. thaliana* and *Ny. colorata*

### 2.3. Gene Structure and Motif Analysis

**Figure 2:**
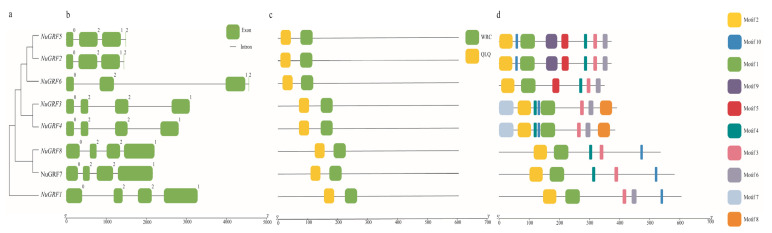
Phylogenetic tree, gene structure, conserved domains, and conserved motifs of GRF genes. (**a**) Phylogenetic tree of NuGRFs. (**b**) Gene structure of NuGRFs, with black lines and green blocks representing upstream or downstream untranslated regions (UTR) and exons, respectively. (**c**) Conserved domains of NuGRFs. (**d**) Predicted motifs of NuGRFs.

Exon–intron structure is one of the essential evolutionary features of genes, and it can provide critical clues about functional diversification. Therefore, we analyzed the exon–intron organization of NuGRFs. The results showed that all NuGRFs contained four exons and three introns (Figure 2b), and the same clade’s NuGRFs had the same lengths of exons and introns (Figure 2a,b), indicating the highly conserved exon–intron structure of *N. nucifera*. Analysis of the conserved domain of GRF genes in *N. nucifera* was performed by NCBI batch CDD. The results showed that all NuGRFs contained the QLQ and WRC conserved domains (Figure 2c). To further understand the structural characteristics of the eight GRFs for *N. nucifera*, the MEME website was used to analyze the conserved motifs of NuGRFs. Ten highly conserved motifs were identified (motifs 1–10) (Figure 2d). The results also showed that the motifs of the GRF gene family belonging to the same clade were conserved in *N. nucifera* (Figure 2a,d). All the NuGRFs had motifs 1, 2, and 3; seven NuGRFs contained motif 10; six NuGRFs contained motif 6, and five NuGRFs contained motif 4. These constitute the typical conserved regions of the GRF gene family. Motifs 7 and 8 existed only in Clade C (*NuGRF3* and *NuGRF4*), and motif 9 was unique to Clade F (*NuGRF2* and *NuGRF5*).

**Figure 3:**
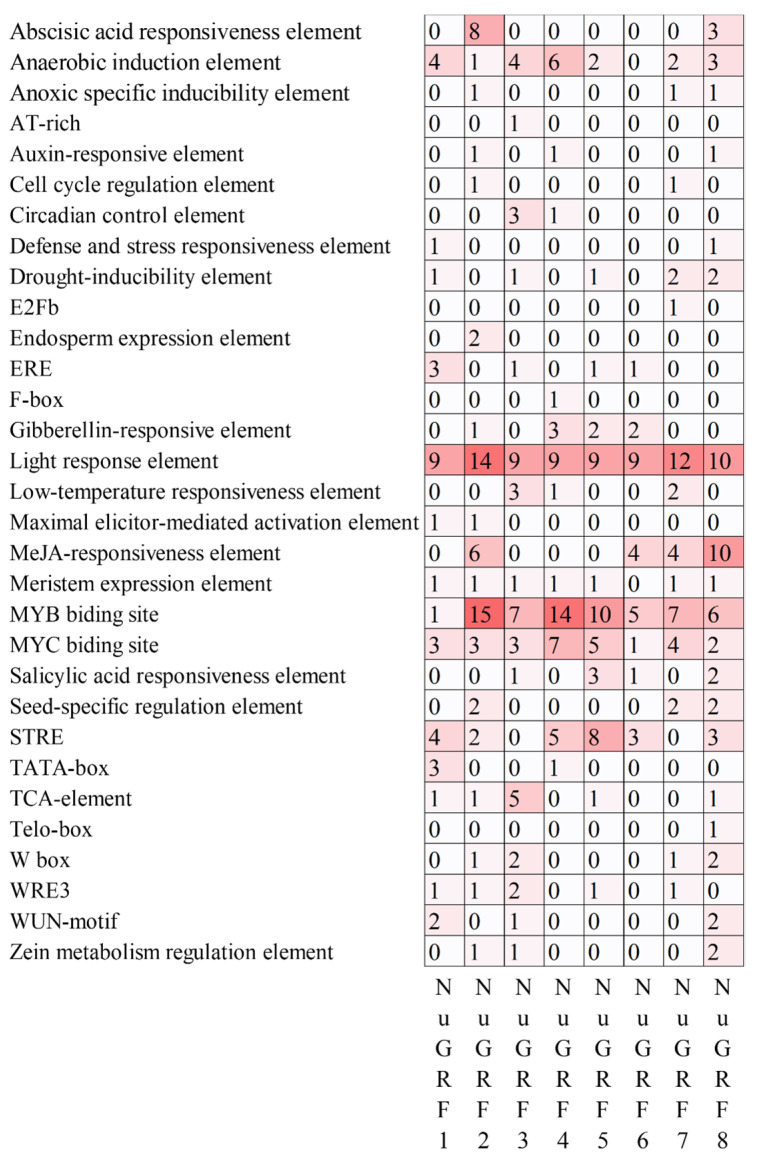
Analysis of cis-acting elements in the promoter regions of GRF genes. The degree of red color represents the number of cis-elements upstream of the NuGRFs; the numbers on the grid represent the number of cis-elements upstream of the NuGRFs.

The prediction results of cis-acting elements of the upstream 2000 bp sequence of the *N. nucifera* GRF gene family showed that NuGRFs contain several TATA boxes and CAAT boxes, indicating that they can be transcribed normally. Moreover, there were a large number of cis-regulatory elements in the promoters of NuGRF genes, among which light response elements (81), MYB binding sites (65), MYC binding sites (28), STREs (25), methyl jasmonate response elements (24), and anaerobic induction elements (22) were the most abundant (Figure 3). All NuGRFs contained an anaerobic induction element, light response element, MYB binding site, and MYC binding site. The main factors related to the growth and development of *N. nucifera* included the cell cycle regulation element, circadian control element, endosperm expression element, seed-specific regulation element, and zein metabolism regulation element. In addition, a large number of hormone-related responsive elements and stress-related cis-acting elements were found in the promoter domain of the NuGRF family. The main elements were auxin-responsive elements (3), defense and stress responsiveness elements (2), drought-inducibility elements (7), light response elements (6), low-temperature responsiveness elements (81), and WUN motifs (5). The results indicate the potential role of GRFs in the growth and development and various hormones and stress-related responses of *N. nucifera*

### 2.4. Chromosome Location and Collinearity Analysis

**Figure 4:**
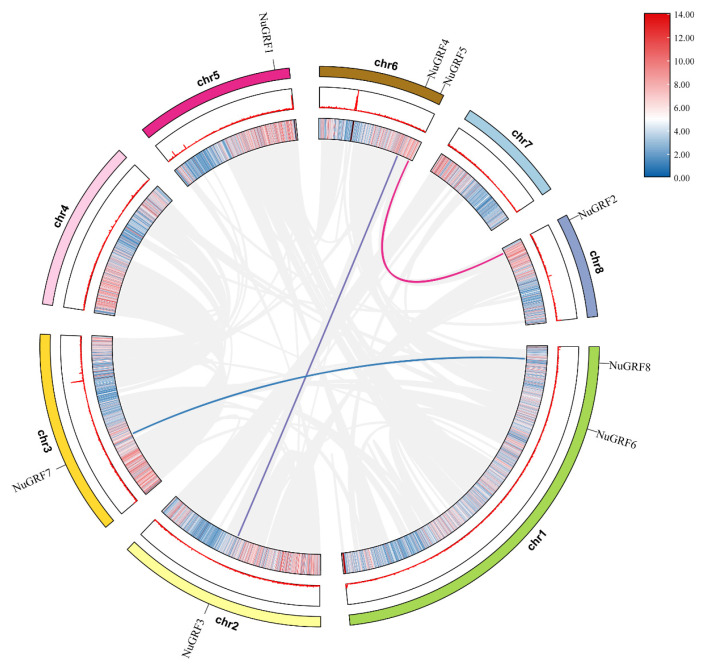
Chromosome distribution and microsynteny relationships among NuGRFs. Lines denote syntenic GRF gene pairs of *N. nucifera* on the chromosomes. ch1~ch8 represent the eight chromosomes of *N. nucifera*.

The eight NuGRFs were unevenly distributed among the eight chromosomes of *N. nucifera* (Figure 4). Chromosomes 1 and 6 contained two NuGRF members, respectively. Chromosomes 2, 3, 5, and 8 contained one NuGRF gene, respectively. Chromosomes 4 and 7 did not include any NuGRF members. Gene replication plays a vital role in plants. We found three gene replication events in *N. nucifera* chromosomes.

**Figure 5:**
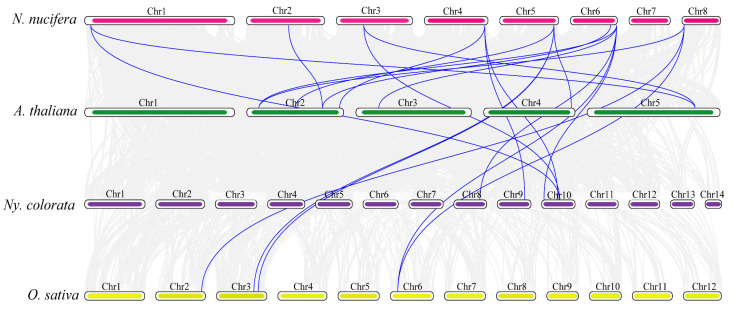
Synteny analysis of GRFs between *N. nucifera*, *A. thaliana*, *O. sativa*, and *Ny. colorata*. The gray lines in the background represent the colinear gene pairs in *N. nucifera* and other species; the blue lines represent the colinear GRF gene pairs.

To further understand the evolutionary mechanism of NuGRFs, collinear analysis was carried out between *N. nucifera*, *A. thaliana*, *O. sativa*, and *Ny. colorata*. The results showed that a total of nine collinear GRF gene pairs of *N. nucifera* and *A. thaliana* were identified, followed by *N. nucifera* and *Ny. colorata* (six pairs), *N. nucifera*, and *O. sativa* (five pairs) (Figure 5).

### 2.5. Expression Pattern of GRF Genes in N. nucifera

**Figure 6:**
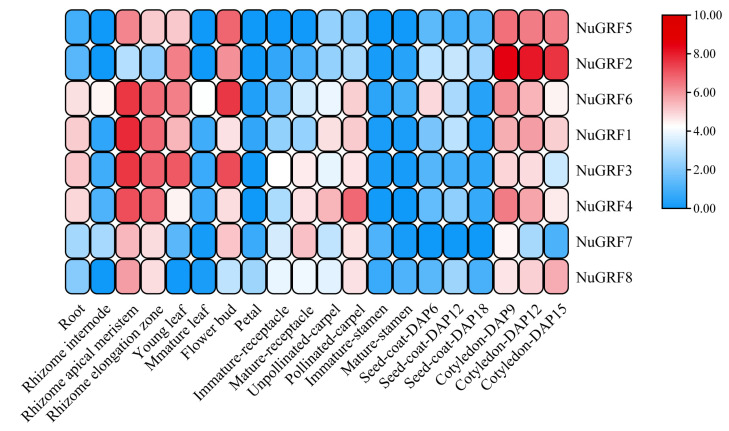
Expression heat map of NuGRFs in *N. nucifera*. Seed-coat-DAP6, 6-day seed coat; Seed-coat-DAP12, 12-day seed coat; Seed-coat-DAP18, 18-day seed coat; Cotyledon-DAP9, 9-day cotyledon; Cotyledon-DAP12, 12-day cotyledon; Cotyledon-DAP15, 15-day cotyledon. The color scale at the right of the heatmap refers to the relative expression level, and the color gradient from faint yellow to dark blue represents increasing expression level.

In order to explore the role of NuGRF family genes in the development of *N. nucifera*, the gene expression matrix of various organs, including different rhizome tissues, roots, leaves, petals, and the receptacle and carpel of different stages of ‘China Antique’ was downloaded from the Nelumbo Genome Database to study their expression patterns. The results showed that the expression of NuGRFs varies in the different tissues of *N. nucifera* (Figure 6). Most NuGRF family genes (except *NuGRF2*) were highly expressed in the differentiated tissues of the rhizome (apical meristem and elongation zone) of *N. nucifera*. All NuGRFs showed low or nearly no expression in the two stages of the stamen. Only *NuGRF6* showed medium expression in the rhizome and leaf, the other NuGRFs showed nearly no expression in these two tissues. Four NuGRFs (*NuGRF1*, *NuGRF3*, *NuGRF4*, and *NuGRF6*) showed medium expression in the root. Except for NuGFR8, which showed low expression in the petal, the other seven NuGRFs showed no expression. *NuGRF3*, *NuGRF4*, *NuGRF6*, *NuGRF7*, and *NuGRF8* showed medium expression in the immature receptacle and were highly expressed in the mature receptacle. *NuGRF1*, *NuGRF3*, *NuGRF4*, *NuGRF6*, *NuGRF7*, and *NuGRF8* showed significantly high expression in the pollinated carpel, while only *NuGRF1* and *NuGRF4* showed high expression in the unpollinated carpel. Except for *NuGRF8*, the other seven NuGRF genes were highly expressed in the flower bud. The NuGRFs were highly expressed in the cotyledon and their expression decreased as the cotyledon developed. Our result showed that the GRF family genes may play an essential role in the development of *N. nucifera*.

**Figure 7:**
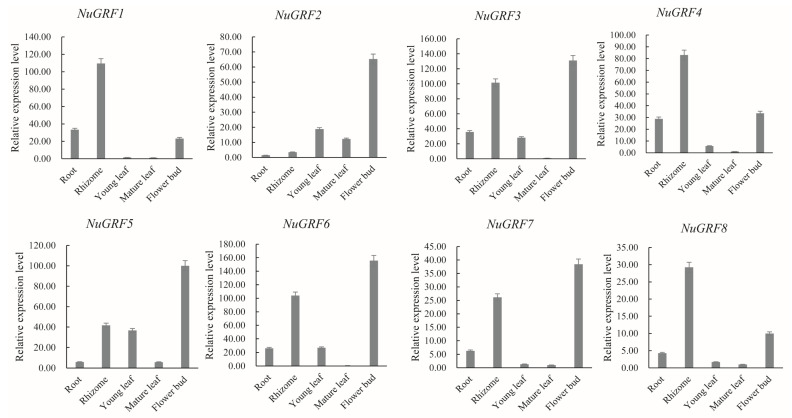
Expression profiles of different tissues of NuGRFs by qRT-PCR.

Then, qRT-PCR was performed to determine the expression pattern of NuGRF genes. The results showed that the qRT-PCR expression of most NuGRF genes (Figure 7) was consistent with the overall trend of transcriptome expression patterns (Figure 6), which further confirmed their preferential expression. The eight NuGRFs were highly expressed in the rhizome and the flower bud. *NuGRF1*, *NuGRF3*, *NuGRF4*, and *NuGRF6* showed medium expression in the root. *NuGRF2*, *NuGRF3*, and *NuGRF6* showed medium expression in the young leaf. The expression levels of most NuGRFs were too low to be detected in the mature leaf, which was consistent with the results from the RNA-seq analysis.

### 2.6. Homology Modeling

**Figure 8:**
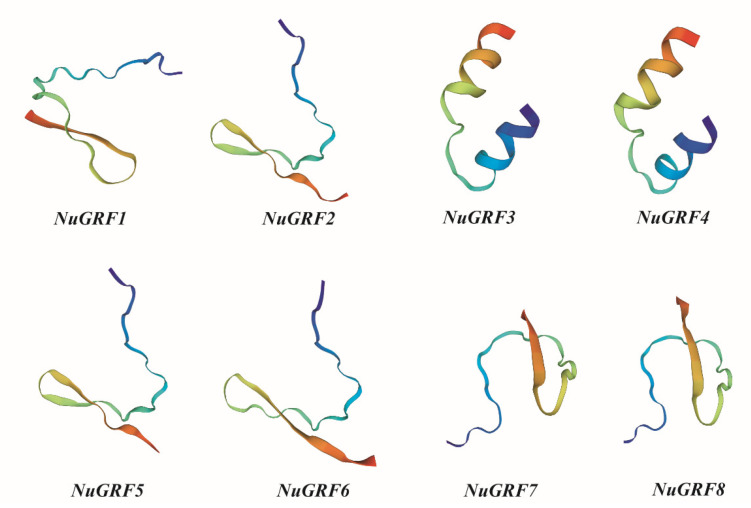
The 3D structure modeling of NuGRF proteins. The tertiary structures were colored by rainbow order, representing N to C terminus.

The SOPMA website was used to predict the secondary structure of NuGRF proteins. The results showed that the NuGRF proteins were mainly composed of random coils (67.29–75.17%), followed by alpha helices (15.59–18.46%), extended strands (5.44–11.96%), and beta turns (0.99–3.18%) (Table 2). The 3D structures of the eight NuGRF proteins were further predicted using the SWISS-MODEL Interactive Workspace website. The results showed that the modeled 3D structures of most NuGRF proteins with respect to the corresponding homologous templates upon superposition had a root-mean-square deviation (RMSD) of less than 1 Å, which indicated that the prediction was reliable. The 3D structure models of NuGRF proteins belonging to the same clade were completely identical (Figure 8). The 3D structure model of the NuGRF proteins plays an important role in underlying their biological functions.

### 2.7. Subcellular Localization Analysis of NuGRF1

**Figure 9:**
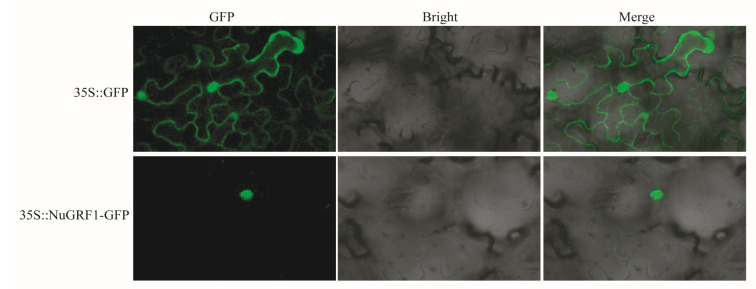
Subcellular localization of *NuGRF1* in tobacco leaf. Scale bars = 10 µm. 35S::GFP was used as the empty control.

The more highly expressed gene, *NuGRF1*, was chosen to assess subcellular localization. We constructed one fusion vector and then transformed it into a tobacco leaf. The results showed that 35S::*NuGRF1*-GFP was located in the nucleus (Figure 9), which was the same as the predicted results (Table 1). In addition, the control group, 35S::GFP, was localized in the nuclear and cell membranes.

## 3. Discussion

The GRF family is a family of important transcription factors. It mainly regulates plant cell size; participates in chloroplast proliferation and gynoecium development; and regulates plant growth and development processes, such as osmotic stress [14,20,30]. Eight GRF genes were identified in *N. nucifera*, less than the number identified in *A. thaliana* (9), *Ny. colorata* (9), citrus (9), and *O. sativa* (12), indicating that the GRF genes may have undergone continuous pedigree-specific expansion and copy loss during evolution [5,9,11].

The phylogenetic analysis indicated that the members of the GRF family could be divided into six clades, and the NuGRFs were only clustered into five clades. This may be due to the different patterns of gene contraction and evolution in plants. The NuGRF genes in the same clade (Figure 1) had the same gene structures and motifs (Figure 2), indicating the functional similarity of GRFs in the same clade [5,9,11]. Comparative analysis of the conserved structural domains showed the presence of complete QLQ and WRC domains in all the NuGRF genes, indicating the high conservation of the GRF family during evolution.

Synteny analysis of NuGRF genes revealed that three gene pairs were syntenic. The collinearity analysis showed that there was a strong linear homologous relationship between *N. nucifera* and *A. thaliana*, followed by that between *N. nucifera* and *Ny. colorata* and that between *N. nucifera* and *O. sativa.*

Previous studies have indicated that the GRF gene family is involved in hormone signal transduction. For example, GRFs are involved in the brassinosteroid (BR) pathway, regulating nuclear development in *A. thaliana* [31]. The GRF genes of *Nicotiana tabacum* are involved in the regulation of gibberellin (GA) biosynthesis [15]. Through the analysis of the cis-acting elements, many hormone-related elements were found in NuGRFs, suggesting a role of NuGRFs in hormone signal transduction. We found many stress response elements in the cis-acting elements of *N. nucifera*, which is consistent with the fact that the GRF gene family plays an important role in stress responses [32,33].

In addition, the GRF gene family plays an important role in the development of organs [11,20,30]. Some cis-acting elements related to development, such as cell cycle regulation, endosperm expression, and seed-specific regulation, were found in the NuGRFs. Previous studies have indicated that the GRF genes are expressed in different parts of the roots, shoots, and flower buds, especially in growing zones where cell proliferation occurs [3,5,9]. Our transcriptome expression analysis showed that NuGRFs were highly expressed in actively growing tissues, such as the rhizome apical meristem, rhizome elongation zone, cotyledon, young leaf, and buds, where cell proliferation takes place vigorously. Previous studies have established that all GRF genes are involved in leaf development [3]. Our study suggests that NuGRF genes play roles in the growth of leaves, and *NuGRF1*, *NuGRF2*, *NuGRF3*, *NuGRF4*, *NuGRF5*, and *NuGRF6* may play more important roles in leaf expansion. Furthermore, GRF gene expression levels in *A. thaliana* decrease as the age of the plant increases [3,16]. NuGRF genes were more highly expressed in young leaves than in mature leaves, and the expression levels of NuGRF genes decreased with the development of the cotyledon, which is consistent with their reported function in the early stages of growth and development in different organs [3,16]. miR396 plays a vital role in flower development in *A. thaliana*. It determines petal characteristics by regulating GRF transcription levels [34]. Overexpression of Ath-miR396a in *A. thaliana* may delay the development of the plant and may cause some specific changes during the flower development, including the third and fourth whorls of floral organs turning into anthers and carpels, respectively [2,34]. In this study, *NuGRF1*, *NuGRF3*, *NuGRF4*, *NuGRF6*, *NuGRF7*, and *NuGRF8* were highly expressed in the receptacle and carpel, suggesting that NuGRFs may play an important role in regulating the development of floral organs. These results were further confirmed in the qRT-PCR.

The 3D structures of the GRF proteins in the same clade were relatively conserved, which was the same for the gene structure, conserved domain, expression pattern, and phylogenetic analysis. The model structure analysis may provide vital clues for better understanding the biological function of the *N. nucifera* GRF gene family. In the end, one fusion vector of *NuGRF1* was constructed and transformed into a tobacco leaf, and the results were in line with the prediction by the website.

In this study, we identified and characterized the *N. nucifera* GRF gene family and evaluated their gene structures, conserved motifs, cis-acting regulatory elements, phylogenetic relationship, chromosome location and collinearity, and expression patterns. These results provide an underlying foundation and framework for further study of the potential physiological roles of each NuGRF gene in the development of the leaf and flower in *N. nucifera.*

## 4. Materials and Methods

### 4.1. Identification of GRF Gene Family

*N. nucifera*, *A. thaliana*, *O. sativa*, and *Ny. colorata* genomics data were downloaded from the Nelumbo Genome Database (http://nelumbo.biocloud.net, accessed on April 2022) [35] and the National Center for Biotechnology Information (NCBI, https://www.ncbi.nlm.nih.gov/, accessed on 1 April 2022). The protein sequences of the GRF gene family of *A. thaliana* and *O. sativa* were downloaded from the TAIR database (http://www.Arabidopsis.org/, accessed on 1 April 2022) and NCBI (https://www.ncbi.nlm.nih.gov/, accessed on 1 April 2022), respectively. The GRF protein sequences of *A. thaliana* and *O. sativa* were used as query sequences to identify the putative GRF proteins in *N. nucifera* and *Ny. colorata* through a local BLASTP program in Tbtools (<le-5) [36]. Then the NCBI Batch CD-search was used to predict whether the candidate gene contained a typical QLQ/WRC domain, and protein sequences with errors or lacking the QLQ/WRC domain were removed. The ExPasy website (https://web.expasy.org/protparam/, accessed on 1 April 2022) was used to predict the protein physicochemical parameters [37], and subcellular localization was predicted using CELLO v2.5 software (http://cello.life.nctu.edu.tw/, accessed on 1 April 2022).

### 4.2. Phylogenetic Relationship Analysis

MEGA7.0 was used for the GRF protein sequences of *N. nucifera*, *A. thaliana*, *O. sativa*, and *Ny. colorata* [38]. The phylogenetic analyses were performed using maximum-likelihood (ML) methods. The ML analysis was performed using the CIPRES Science Gateway web server (RAxML-HPC2 on XSEDE 8.2.10) with the following settings: sampling frequency = 1000; tem = 0.1; burn-in = 2000; and number of Markov chain Monte Carlo generations = 10,000,000 [39].

### 4.3. Gene Structure, Conserved Motifs, and Cis-Acting Element Analysis

The exon–intron structures of NuGRFs were displayed by Tbtools [36]. Multiple Expectation maximization for Motif Elicitation (MEME, http://meme-suite.org/, accessed on 1 April 2022) was used to analyze the conserved motifs of each NuGRF gene protein [40]. The upstream 2000 bp sequences of NuGRF genes were extracted as promoter sequence information, and the cis-acting elements of the GRF gene of *N. nucifera* were predicted by the online prediction software PlantCare [41]. The cis-acting elements were enriched and visualized by Microsoft Excel 2010.

### 4.4. Chromosome Location and Collinearity Analysis

The intraspecific and interspecific collinear relationships of GRF families were obtained using the One-step MCScanX function of TBtools, and the intraspecific and interspecific results were visualized by Advanced Circos and Multiple Synteny Plot of TBtools software, respectively [36].

### 4.5. Expression Pattern Analysis

The gene expression matrix of different organs (young leaf, mature leaf, flower bud, rhizome internode, rhizome apical meristem, rhizome elongation zone, petal, immature receptacle, mature receptacle, unpollinated carpel, pollinated carpel, mature stamen, immature stamen, and seed coat and cotyledon on different development days) of ‘China Antique’ was downloaded from the Nelumbo Genome Database (http://nelumbo.biocloud.net, accessed on 1 April 2022) [35]; the expression levels of NuGRFs were calculated as fragments per kilobase of exon per million fragments mapped (FPKM), the average of multiple samples was calculated, and TBtools was used to visualize the heatmap [36].

To verify the expression pattern of GRF genes, the root, young leaf, mature leaf, and flower bud were sampled from *N. nucifera* that were planted at Fujian Agriculture and Forestry University for RT-qPCR analysis. Each organ was sampled in three replicates. The total RNA was extracted using the FastPure ^®^ Plant Total RNA Isolation Kit (Polysaccharides and Polyphenolics-rich) (Vazyme, Nanjing, China). The concentration of total RNA was determined by a Nanodrop 2000 spectrophotometer, and the integrity of total RNA was detected by agarose gel electrophoresis. The RNA was reverse transcribed into cDNA using the PrimeScript^RT^ reagent Kit with the gDNA Eraser (CodeNo.RR047A) kit. The RT-qPCR primers were designed by the Primer3Plus online tool (Table S1). The Taq Pro Universal SYBR qPCR Master Mix kit (Vazyme, Nanjing, China) was used for qRT-PCR. The relative expression of genes was calculated by the 2^−^^ΔΔ^^Ct^ method.

### 4.6. Protein Structure Prediction

The secondary structure of the NuGRF protein sequence was predicted by the online software SOPMA [42]. The 3D structure of the protein was predicted by the online SWISS-MODEL Interactive Workspace, selecting the template with a consistency of more than 30% with the NuGRF protein sequence [43].

### 4.7. Subcellular Localization Analysis

The RNA was extracted from the mixed organs (root, young leaf, and flower bud). Then, the RNA was reverse transcribed into cDNA. The primers of the *NuGRF1* coding sequence (CDS) without the stop codon were designed by Snapgene 3.2.1. The primers of *NuGRF1* used in this study were as follows: forward: gagaggacctcgactATGGATTTTGGAGTGGTAGGTTTG, reverse: tttttctaccggtacCAATGAAGGGATGGAGGAGGA. PCR amplification was performed using Phanta Super-Fidelity DNA Polymerase (Vazyme, Nanjing, China) with cDNA as a template. Then, the *NuGRF1* coding sequence was cloned into the pMDC202 vector, which contains a 35s-driven green fluorescent protein (GFP) promoter, using the In-Fusion Cloning Kit (ClonExpress^®^ Ultra One Step Cloning Kit, Vazyme, Nanjing, China). The restriction sites were XbaI and KpnI. Moreover, the 35S::*NuGRF1*-GFP was transformed into a tobacco leaf, and the vector without a gene was used as a control. The transformed tobacco was cultured first in the dark for eight hours at 23 °C and then in light for 16 h at 25 °C. After 48 h, the GFP fluorescence signals were observed using an LSM710 confocal laser scanning microscope (CarlZeiss, Jena, Germany).

## 5. Conclusions

In this study, the GRF gene family was identified for the first time in the whole genome of *N. nucifera*. On the basis of the phylogenetic relationship, eight genes were divided into five clades. Then, gene structure, conserved motifs, cis-acting element, chromosomal gene distribution, and collinearity analyses were performed. Subsequently, we combined the expression data and RT-qPCR data and speculated that the NuGRFs might play a regulatory role in the development of actively growing *N. nucifera* tissues, such as the young leaf and flower bud. The secondary and tertiary structures of the NuGRF protein were predicted. Finally, we performed subcellular localization analyses for *NuGRF1*. These results provide a reference for further study of the function of NuGRF genes in the regulation of flower and leaf development and the genetic improvement of *N. nucifera* flower type.

## Figures and Tables

**Table 1:** Characteristics of the GRF genes in *N. nucifera*.

Gene	Number of Amino Acids	Molecular Weight (Average)	PI	Instability Index	Aliphatic Index	Grand Average of Hydropathicity (GRAVY)	Subcellular Localization
*NuGRF1*	604	65,565	8.14	58.7	65.71	−0.579	Nuclear
*NuGRF2*	372	42,187.6	8.61	64.37	50.4	−0.881	Nuclear
*NuGRF3*	390	42,189.9	8.7	55.67	62.59	−0.602	Nuclear
*NuGRF4*	384	41,746.4	8.92	55.27	63.52	−0.605	Nuclear
*NuGRF5*	372	42,107.57	8.95	57.59	52.98	−0.902	Nuclear
*NuGRF6*	349	38,940.19	8.47	59.49	47.28	−0.762	Nuclear
*NuGRF7*	581	61,707.56	6.36	57	53.96	−0.492	Nuclear
*NuGRF8*	535	535	8.39	65.39	65.39	−0.455	Nuclear

**Table 2:** Secondary structure of NuGRF proteins.

Gene	Alpha Helix (Hh)	310 Helix (Gg)	Pi Helix (Ii)	Beta bridge (Bb)	Extended Strand (Ee)	Beta Turn (Tt)	Bend Region (Ss)	Random Coil (Cc)	AMBIGUOUS States (?)	Other States
*NuGRF1*	14.74%	0.00%	0.00%	0.00%	9.11%	0.99%	0.00%	75.17%	0.00%	0.00%
*NuGRF2*	15.59%	0.00%	0.00%	0.00%	10.75%	2.96%	0.00%	70.70%	0.00%	0.00%
*NuGRF3*	18.46%	0.00%	0.00%	0.00%	11.03%	2.05%	0.00%	68.46%	0.00%	0.00%
*NuGRF4*	17.19%	0.00%	0.00%	0.00%	10.42%	2.08%	0.00%	70.31%	0.00%	0.00%
*NuGRF5*	15.59%	0.00%	0.00%	0.00%	10.75%	2.96%	0.00%	70.70%	0.00%	0.00%
*NuGRF6*	17.19%	0.00%	0.00%	0.00%	5.44%	3.15%	0.00%	74.21%	0.00%	0.00%
*NuGRF7*	17.38%	0.00%	0.00%	0.00%	11.19%	2.93%	0.00%	68.50%	0.00%	0.00%
*NuGRF8*	17.57%	0.00%	0.00%	0.00%	11.96%	3.18%	0.00%	67.29%	0.00%	0.00%

## Data Availability

Not applicable.

## Supplementary Materials

The following supporting information can be downloaded at: https://www.mdpi.com/article/10.3390/ijms23126673/s1.
