# Supplementary material for: Comprehensive Analysis for GRF Transcription Factors in Sacred Lotus (Nelumbo nucifera)
Source: Int J Mol Sci. 2022 Jun 15;23(12):6673. doi: 10.3390/ijms23126673 (PMC9224289; doi:10.3390/ijms23126673)
Supplement: Supplementary file 1 [file ijms-23-06673-s001.zip › ijms-1747634-supplementary.pdf]

Table S1. The primers for RT-qPCR, the NuACT was the reference gene.

Gene	Primer	Sequence(5'to3')
NuGRF1	NuGRF1-F	AGACCCAGTACACTAGGATGG
	NuGRF1-R	TCTTGAGCACCGCCATTTCT
NuGRF2	NuGRF2-F	CCCACCGGATCTCATCTTTCC
	NuGRF2-R	CCCACCCAATAGGTTGGTGA
NuGRF3	NuGRF3-F	ATGAACTGGGCATCAGGGTG
	NuGRF3-R	CTCATGCAGGACGCTTGTTG
NuGRF4	NuGRF4-F	CTTGACAGCGGTGGCTAGTG
	NuGRF4-R	ACCAGTACCTTCTGAGGATCTTT
NuGRF5	NuGRF5-F	AGAGACACATGCACAGAGGC
	NuGRF5-R	AGGGAGTAAGAAGAGGCGGT
NuGRF6	NuGRF6-F	GACTGCCTGTCCCTCCTGA
	NuGRF6-R	AGAAGGAACAATAACCCATGCTG
NuGRF7	NuGRF7-F	CCAATCTACCATGGGTCGGG
	NuGRF7-R	GAACACCGCCACTTCTTTCC
NuGRF8	NuGRF8-F	ATATTCGATGCCGTTGCTGGA
	NuGRF8-R	GCAGCCATCCCTTCTGGG
NuACT	NuACT-F	TGGCAGACAACGAGGATATTC
	NuACT-R	CTACAATGCTAGGGAACACGG
